# Ierne Finds a Task for Every Member of the College

**Published:** 1919-07-19

**Authors:** 


### PROPAGANDA.

### Ierne Finds a Task for Every Member of the College.

PROPAGANDA, as a science or an art, whichever it may be, is a war product. Germany had taken to it with characteristic thoroughness long before, but it was, all the same, merely a part of her war programme. In every country in the world, savage or civilised, her agents toiled unceasingly and with a common purpose. There were no limits and no scruples. Clerics, journalists, soldiers, princes of the blood, assassins, thieves, women with and without reputation—all these and a score of other types found employment in Germany's propaganda army, which was, despite the variety, by no means nondescript, but magnificently organised, and by a master hand. No reasonable person, indeed, can doubt that the most accomplished master of propaganda is the devil. Leave him and his camp followers out of count, and all the rest of the world are mere *dilettantes.*

### THE CONCEALING CLOAK.

Have you taken account of the Bolshevist, naked and undisguised, or subtly concealed under a cloak of education, culture, and fine manners? He began in Russia, and some people think that he is still there, whereas he is in our midst promoting strikes and mutinies and rebellions. He rides through the London streets in a first-class car, and fares sumptuously every day.

I am not now concerned with German or Bolshevik wickedness, but with their marvellous methods of procedure, their ceaseless activity, ingenuity, determination. No obstacle is too great to surmount, no reverse too serious to cause discouragement. If Christianity had half such zealous agents the dove bearing her olive branch of peace would not find it so difficult to alight. Can we not steal a page from the devil's book, and learn the value of thoroughness? The war has taught us the value of propaganda, but it is, so far, little more than a name in our vocabulary. It is something like flying. We think we know all about flying because we see aeroplanes and airships. But what manner of people should we be if we could all fly, as do the birds? We have not yet even touched the fringe of the flying problem, and in like manner we have been only toying with propaganda. What, for example, has the College of Nursing done by way of propaganda? Probably as much as a great many equally worthy associations, and much more than most young societies established for the welfare of humanity. Hitherto a false idea of dignity, or rather one that is not up to date, has been the restraining influence. Yet, withal, taking account of what might be done, and comparatively speaking, nothing.

Efficient propaganda consists in using to the fullest every opportunity that offers, and creating opportunity where there is none. Masters of commerce do more than supply the public demand. They first cause the demand—call it into existence. So the propagandist has not only to use his opportunities, but to produce them.

### THE PERSONAL FACTOR.

In the early days of its existence the College was a voice crying in the wilderness. It consisted of hopes and dreams preached by a handful of enthusiasts. And it can never be said of these that they lacked efficiency or thoroughness. The fabric that they have builded speaks for itself. But now there is an army numbering upwards of fourteen thousand, each member of which should be a vigorous propagandist. It is due to the founders and builders of the College that every member should be a missioner having a prescribed duty to perform as a labour of love. If this were actually so the College membership would double itself within a month, for it is estimated that there are five times as many trained nurses in the Kingdom as there are members of the College, and every non-member has much to gain and nought to lose by joining. Moreover, those who are already within the fold will profit in manifold degree by adding to their forces. Enemies of the College have abusively described it as a benevolent institution, a registration society, and a university. If all this it appears to the jaundiced eye what must its benefits, actual and potential, be to those who are its members, and in whose hands are its development and its destiny!

### AN ALLOTTED TASK.

There is in propaganda no factor to compare with the individual advocate, and therefore, I submit, every College member ought to have an allotted task, not in general terms, but specific and precise. She ought to be a source of information within her area of operations, an organised entity in a missionary machine, communicating with a local organiser, who would in turn receive direction and inspiration from G.H.Q. There is no reason why the devil should have a monopoly of efficiency and thoroughness. The present is the British nurse's golden opportunity for emancipation and achievement, and every thousand new recruits to the College at this stage of its life and growth substantially helps to bring realisation and possession nearer. The voice of fourteen thousand has already rescued the College from a great tyranny The voice of forty thousand would resound throughout the Empire.

### BENEFIT OF COLLEGE MEMBERSHIP.

What actual and material benefits has College membership to offer? The answer depends altogether on the strength of the membership. So long as this is insignificant the College will be a struggling community fighting its way against apathy and enmity. If it becomes overwhelmingly strong no reasonable demand that its members make can be denied them. Its leaders and governors will be of their own choosing, its policy the expression of their own will. The British nation values and appreciates its nurses more highly than they wot of—but the nation is unable to distinguish between the discordant voices that fill the air. This is, in effect, what the Government states when it declines to accept the College Registration Bill. Even fourteen thousand voices are a small minority.

Is it not possible and desirable in the immediate future to issue a whip to every College member, urging upon her the duty of recruiting. The effect of a large accession of strength to the College within the next month or two must undoubtedly add to the power of its supporters in the House of Commons. I entertain no doubt that if this is done there will be a wholehearted and loyal response.

IERNE.

